# Why should we promote public engagement with science?

**DOI:** 10.1177/0963662513518154

**Published:** 2014-01-16

**Authors:** Jack Stilgoe, Simon J. Lock, James Wilsdon

**Affiliations:** University College London, UK; University of Sussex, UK

**Keywords:** governance, public dialogue, public engagement

## Abstract

This introductory essay looks back on the two decades since the journal *Public Understanding of Science* was launched. Drawing on the invited commentaries in this special issue, we can see narratives of continuity and change around the practice and politics of public engagement with science. Public engagement would seem to be a necessary but insufficient part of opening up science and its governance. Those of us who have been involved in advocating, conducting and evaluating public engagement practice could be accused of over-promising. If we, as social scientists, are going to continue a normative commitment to the idea of public engagement, we should therefore develop new lines of argument and analysis. Our support for the idea of public engagement needs qualifying, as part of a broader, more ambitious interest in the idea of publicly engaged science.

## 1. Introduction

In an influential 1987 article, Thomas and Durant asked a question that remains pertinent today: why should we promote the public understanding of science? (Thomas and Durant, 1987). Five years later, the journal *Public Understanding of Science* (PUS) was launched as a forum for debating this question as it grew in policy and practical significance. This journal has steadily described and mapped the gradual and incomplete shift from ‘understanding’ to ‘engagement’, and the flowering of policies, initiatives and practical experiments that has accompanied this.

The journal’s launch twenty years ago was motivated primarily by ends-based debates about the governance and political legitimacy of science and technology. But such ends-based discussions have since been often overshadowed by a focus on the means and processes of public engagement. The *how* trumps the *why*, and there is insufficient systematic reflection on what all this activity has achieved. We share a normative commitment to the idea of democratic science policy, and have argued that public engagement can be a part of this.

Our motivation in convening this special issue of *Public Understanding of Science* was to reflect on what has changed over two decades, what remains the same and what dimensions of public engagement in science and technology remain neglected or under-examined. As in the first issue of the journal, we have commissioned a set of short perspectives from leading scholars and practitioners, drawn from a range of theoretical, disciplinary and geographical contexts. We asked each of them to reflect on their involvement in research and practice in this field and, most crucially, to look forward and ask where we are going. Why should we continue to argue for public engagement with science?

In this introduction, we reflect on the essays written for this special issue, alongside other recent insights from the literature. Our conclusion is that support for the idea of public engagement needs qualifying, as part of a broader, more ambitious interest in the idea of publicly engaged science.

## 2. Continuity and change

Each of the contributors to this special issue points to dynamics of change and continuity. The move from ‘deficit to dialogue’ is now recognised and repeated by scientists, funders and policymakers. Social scientists and engagement practitioners have also announced this move, for reasons that are sometimes analytical and sometimes rhetorical. But for all of the changing currents on the surface, the deeper tidal rhythms of science and its governance remain resistant. Several of the essays here follow earlier criticisms of the way in which dialogue continues to reflect deficit-like assumptions (Grove-White, 2001; Rayner, 2004; Wynne, 2005).

The most visible manifestation of the move from deficit to dialogue has been the proliferation of various dialogue processes. Understandably, for those interested in public engagement with science, these ‘mini-publics’ (Goodin and Dryzek, 2006) have been put under the spotlight as interesting experiments or perhaps performances in governance (Stilgoe, 2012, after Collins, 1988). There is a growing recognition, represented in the essays in this issue, that too much analysis has focused on the wrong level of experimentation. We need to take a step back and view engagement in its wider political context.

Several of the contributors to this collection (Michael Burgess, Dave Guston, Richard Jones) describe particular examples of public dialogue on issues that are in some way scientific. In the decade or more since public dialogue became a normal part of the science governance landscape, we have become used to seeing descriptions of such exercises, alongside evaluations and critiques. Patrick Sturgis expands on some of these in his essay here. Critiques tend to focus on the engagement exercises themselves, and the questions about legitimacy that they ask relate to inputs – what goes into engagement. In the way engagement is currently practised, Lövbrand et al. (2011: 483) suggest that there is a “fundamental problem of scale”; processes seem legitimate only for the people who are involved in them. For those interested in broader questions of science and democracy, this would seem to be a fundamental problem, unless we take a wider view of the governance experiment of which engagement is a part.

Irwin et al.’s (2013) analysis of discussions that take place around public dialogue suggests that we should learn the common moves practised by proponents and opponents of dialogue so that we become familiar with them and engage in the ‘higher order game’ (Irwin et al., 2013: 131), of which individual dialogue exercises form only a part. So once we understand that particular engagements will inevitably be met with questions of representativeness, articulation, impacts and outcomes, we can look at what else is going on, how the tone of voice and ‘body language’ (Wynne, 1996; Grove-White et al., 2000) are changing. Taking this argument into evaluation practice, therefore, we can see the limits of evaluating individual exercises in their own terms. We share the concerns of O’Doherty and Einsiedel (2012), also expressed by Alan Irwin in his essay here, that the literature risks becoming a litany of engagement case studies and evaluations.

As public money and legitimacy is invested in dialogue processes, it is right to ask about their quality. But the procedural demands of engagement funders are not the only, nor the most important, considerations. Just as more scientific evidence does not win political battles on issues such as climate change (Sarewitz, 2004), so the quest should not be for internal perfection in engagement processes. We can instead point to the value of critical, evaluative research that looks not at particular dialogues, but at the broader project of dialogic governance (see, for example, Macnaghten and Chilvers, 2013). Such research has challenging and important implications, because it includes the institutions that support public engagement as part of the experimental apparatus.

The legitimacy of public engagement does not just depend on its inputs, but also on its outputs (van Oudheusden, 2011, after Scharpf, 1999), in particular its impact on governance. The suspicion is that such exercises do not sufficiently challenge, and so serve to reinforce, incumbent power structures. Public engagement can be seen by institutions as an opportunity not to rethink their policies and practices, but to gain trust for a predetermined approach (Wynne, 2006; Thorpe and Gregory, 2010). When institutions initiate dialogue, what might be envisaged as a potential ‘technology of humility’ (Jasanoff, 2003), capable of prompting institutions to question their governance, can become a ‘technology of elicitation’ (Lezaun and Soneryd, 2007), extracting public opinion in convenient ways. Rather than opening up decision-making, public dialogue might be implicated in its closure, by preventing alternative views from surfacing (Stirling, 2008; Chilvers, 2010; Sturgis, this issue). Too often, the emphasis is on ‘consensus’, which disguises the diversity of views that are likely to define a particular issue (Mohr, 2008).

The more public engagement is practised, the clearer becomes the tangle of institutional motivations behind it. Helga Nowotny, in her essay, makes a strong argument that, despite the “tacit understanding … that public engagement with science had to do with linking science to politics”, there has been a collective failure to take politics seriously. Similarly, as Richard Jones argues in his essay, public engagement has become proceduralised and we have at times lost sight of the problems to which it is offered as a solution. Both Burgess and Guston offer an optimistic account of public engagement, suggesting that, if decision-makers are themselves enrolled in dialogue, and if connections are made to politics, these processes can not only succeed in their own terms, but generate further conversations and capacities that move us some way towards what Guston describes as “a revival of how we conceive of science in our politics”. This means also paying attention to the political economy and ‘de facto governance’ of science (Kearnes and Rip, 2009; Lave et al., 2010). Public engagement may be a necessary but insufficient part of investigating these dynamics.

The institutionalisation of public engagement invites an important new set of considerations. We cannot continue to pretend that ‘talk is cheap’. Meaningful dialogic public engagement is both costly and politically risky (Kleinman et al., 2011). Expectations, which, as we describe above, can be confused, are nevertheless increasing. As public dialogue has become institutionalised, critical academics are joined by practitioners and policymakers who may be more interested in efficiency than reflexivity, and expectations change. In her essay here, Maja Horst points to one institutional case of what appears to be engagement fatigue. The Danish Board of Technology, “more celebrated internationally than it is at home”, according to Horst, now faces harsh scrutiny. The science and technology studies (STS) insight that “distance lends enchantment” (Collins, 1985) in science would seem to apply equally to public engagement.

In the post-financial crisis period of austerity that Nowotny describes, we will hear the question ‘is public engagement worth it?’ increasingly often. This is almost impossible to answer, especially as the most important benefits of engagement may be those that are challenging and inconvenient for the institutions that fund it. Critical scholars of engagement have pointed out that engagement exercises do not seem to be delivering on their promises (see, for example, Scheufele, 2011). But even if such criticisms are correct, productive engagement exercises typically open up areas of dissensus, and can generate further questions about trust. The ‘social intelligence’ generated may be challenging or irrelevant to the particular institutions invested in the exercise, but nevertheless important (Stilgoe, 2007). By ‘overflowing’ (Callon et al., 2009) the remits set for them, public engagement exercises can have all sorts of other benefits (a point developed by Burgess in his essay). There would seem to be an urgent need to develop criteria for mapping and evaluating these spillovers. Making sense of the unintended (at least for the people who pay for it) consequences of engagement represents an important task for social scientists.

## 3. Imagining the public

As Sturgis describes in his essay, our models of engagement often appear to be based on half-baked ideas of deliberative democracy and the public. Sturgis suggests that “we know rather little about whether the public are as keen on participatory dialogue as those who advocate it as key to democratic governance”. We need to know more about fatalism with respect to science governance and disenchantment about engagement, and question the constructed publics that are being invoked in the discourse and practice of engagement.

A clear achievement of more than two decades of research into public engagement has been to shift attention to the ways in which ‘the Public’ has been constructed in public engagement. A public imagined as ignorant and hostile was the impetus for many of the science communication activities in the 1980s and 1990s, and though this transformed into more sophisticated ideas of engagement with multiple ‘publics’ for science and technology, such publics were often still imagined as ‘concerned’, ‘anti-scientific’ or ‘obstructions’ to innovation (House of Lords, 2000; Owens, 2000). As Barnett et al. (2012: 47) have recently argued, “the construction and expert control of public concern invites interactions framed in terms of expert reassurance rather than mutual exchange and engagement”. There is a sense among institutions that have experimented with engagement that exercises are hampered by publics that are far from ‘ideal’ citizens (Lezaun and Soneryd, 2007). Stage-managed spaces of engagement preclude the potential for ‘uninvited publics’ to engage with science and technology and widen the interaction and scope for reflexivity (Wynne, 2011). When such engagements take place, outside the penumbra of organised dialogue, we are quickly reminded, as Sheila Jasanoff reminds us in this issue with her account of “Science: it’s a girl thing”, of the institutional propensity for “hitting the notes, but missing the music” (Wynne, 2006).

## 4. Where next?

### Emerging agendas of responsible innovation

A consequence of the dynamics described in this special issue is that public engagement has become a means in search of an end. Confusion or deliberate obfuscation of broader political discussion in an attempt to make public engagement procedurally comfortable has meant that the deficit models we thought were dead are continually reinvented (Rayner, 2004; Bauer et al., 2007). The much-touted move from deficit to democracy has perhaps been hampered by a continued focus on The Public. It has been relatively easy to make the first part of the argument that monologues should become conversations. It has been harder to convince the institutions of science that the public are not the problem. The rapid move from doing communication to doing dialogue has obscured an unfinished conversation about the broader meaning of this activity. It is not simply a matter of science providing a microphone as well as a megaphone. The need for institutional reflexivity (Wynne, 1993) fundamentally challenges who should be doing engagement and why.

A new term recently put forward in an attempt to move beyond this pathologising of the public comes with the enthusiasm for ‘responsible innovation’ (or ‘responsible research and innovation’) (von Schomberg, 2011; Stilgoe et al., 2013). This builds on ideas of anticipatory governance, Real-Time Technology Assessment, Constructive Technology Assessment, value-sensitive design and open innovation that all incorporate ideas of public and user engagement (see, variously, Rip et al., 1995; Friedman, 1996; Guston and Sarewitz, 2002; Chesbrough, 2003; Barben et al., 2008). At the European Commission, areas of work that would previously have been called ‘science in society’ are now talked about as ‘responsible research and innovation’. The term has superficial political (and indeed corporate) appeal, which means it runs all the same risks of instrumentalism that ‘public engagement’ has suffered from (Owen et al., 2012).

### Diverse civic epistemologies

As Jasanoff argues in this issue, it is now time to re-open our ideas about publics and science. Publics, she states, “are not all alike but are guided by culturally conditioned ‘civic epistemologies’”. We should think of ‘The Public’ less as a pre-existing entity and more as a space within which publics selectively form around technoscientific objects and matters of concern. It is these issue-oriented publics, Jasanoff contends, who enter the political arena to participate in constructing scientific and technological futures. Crucially, as Wynne also argues here, it is the public meanings attached to science and innovation that should be allowed more space and influence in the political economy of science rather than their being discounted in the face of scientifically-defined problems and risks.

As Helga Nowotny describes, publics can be global consumers of scientific knowledge yet citizens within very local and specific political structures. Public engagement rightly reflects this cultural diversity. In their essay, Hepeng Jia and Li Liu describe “[t]he hard road from science popularisation to public engagement” in China, which, despite very different political institutions and democratic norms from those in Europe and the US, has seen a wave of “pilot projects of public participation in science, including the country’s first pilot consensus conference”. As global concentrations and patterns of scientific activity change, and emerging economies such as China play a more significant role in networks of research and innovation, we can expect distinctive models and approaches to engagement to emerge (Leadbeater and Wilsdon, 2007; Royal Society, 2011). Anticipating and analysing the contours of ‘public engagement with Chinese characteristics’, and its equivalents in India, Brazil, South Africa, Egypt or Indonesia, is likely to be one of the most dynamic and exciting research agendas for public engagement with science over the next twenty years.

### Inconvenient, marginal and emergent domains

Multidisciplinary work on the Ethical, Legal and Social Issues (or Implications) (ELSI) associated with emerging science and technology has prompted criticism from some that social science has been enrolled in potentially damaging ways. The first line of this ‘ELSIfication’ critique (Williams, 2006; Rabinow and Bennett, 2009; Lopez and Lunau, 2012) is that eager social scientists, philosophers and lawyers can lose their critical edge as they become too cosy with scientists. As Langdon Winner argued when giving evidence to the US Congress,

‘I would not advise you to pass a Nanoethicist Full Employment Act, sponsoring the creation of a new profession. Although the new academic research in this area would be of some value, there is also a tendency for those who conduct research about the ethical dimensions of emerging technology to gravitate toward the more comfortable, even trivial questions involved, avoiding issues that might become a focus of conflict’. (Winner, 2003, quoted in Fortun, 2005: 159)

The second critique of ELSIfication is that it contributes to the reification of particular areas of science as important and transformative. Alfred Nordmann (2007) cautions against ‘speculative ethics’. The final, more practical problem is that social scientists, by taking too literally the STS maxim of ‘following scientists around’ (Shapin, 1988), risk defining themselves in scientific terms and allow their studies and engagement to be skewed accordingly. Narratives of hope and fear from scientists and policymakers have led to substantial social research and public engagement around genomics and the life sciences, while domains such as financial innovation and ICTs (information and communication technologies), whose sociotechnical dynamics are more balanced towards the socio, are less intensely scrutinised. If public engagement is to have political purchase, it must happen in places and on issues that are inconvenient, emergent or marginal, as well as those in which scientific communities are well-defined and receptive. As Irwin argues in this issue there can be “a tendency to dump all the difficult socio-institutional challenges into the ‘social science’ basket – thus liberating scientific institutions from their own obligation to take such matters seriously.” It also remains the case that, while most innovation happens in the private sector, public engagement is overwhelmingly focused on universities and other public sector bodies (Wilsdon and Willis, 2004).

### New platforms and spaces for engagement

Much of the new money available for dialogue, at least in Europe, has come from government sources attached to science and technology issues seen as strategically important. Yet over the last twenty years there has been a huge growth in informal engagement activities such as science festivals and online spaces for science communication and engagement. Social media have revealed an enthusiasm for uncontrolled engagement among those interested in science. Less academic attention has been focused on sites of engagement between publics and science outside of the policy setting (Davies et al., 2009; Jensen and Buckley, 2012) and as such, we know little about the rationales, agendas and activities that are in operation in these newer spaces.

It is tempting to criticise informal learning events as peddling deficit model approaches, but evaluations of informal science engagement events have noted the variety of rationales for participation (Davies, 2008; Burchell et al., 2009; Wilkinson et al., 2011; Jensen and Buckley, 2012). Social media have connected previously disparate groups of ‘science enthusiasts’ or so-called ‘geeks’ (Henderson, 2012). However, as yet there has been little research on the motivations of such individuals to engage with each other and why and how in some cases they have moved from informal settings such as pubs, festivals and cafes to effective lobbying on issues such as libel law and science funding.^1^ Such activities break down any clear distinction between informal, policy-free engagements and politically motivated activities. Much has also been made of the growing activity that falls under the wide umbrella of ‘citizen science’ (Ince, 2011; Gura, 2013). Yet much of this activity, even if it takes place outside a formal laboratory, seems to do little more than replicate existing power relationships between scientists and publics (Haklay, 2013). There is much to understand here about these new spaces for engagement with science and technology and their impacts on scientific culture, politics and society. As Nowotny argues in this issue we have “to follow the engagement of citizens with new technologies and how their use of the new media shapes, constrains and possibly widens the choices open for science and democracy.” Likewise, as Horst suggests in the case of Denmark, in trying to institutionalise or ‘tame’ public engagement activity we risk ignoring or discounting places outside of the formally mandated engagement processes where publics do, or wish to, engage with science, technology and innovation.

### Renewed enthusiasm for open science

The openness of science that has been a hallmark since the 17th century now seems to be undergoing a process of reshaping and renegotiation (David, 2008). The growing volume and intensity of debate, activity and advocacy around agendas of open access, open data, and open science has been striking (Nielsen, 2011: 272; Royal Society, 2012; RCUK, 2013; Willetts et al., 2013). Open access is set to become the default mode for scientific publishing in the UK, and potentially across the rest of Europe. The opening up of data has seen arguments about the efficiency of science intertwined with those about the politics of science, as, for example, in the ‘Climategate’ episode.

There are of course real limitations to these moves. For example, open access could, as Richard Jones suggests in his article, become a distraction from, or in some minds a replacement for, the larger challenge of developing meaningful public engagement with research. Likewise, claims for it putting “power in the hands of the public funders” (Willetts, 2012) may be overblown as Steve Fuller has argued (Fuller, 2012). However, this drive for greater openness, albeit limited to certain aspects of the scientific enterprise, is an important contemporary dynamic of science, ripe with opportunity for scholars and advocates of public engagement.

## 5. Conclusion

The pieces in this special issue are all invited contributions. Many of the authors need little introduction. Sheila Jasanoff, Alan Irwin and Brian Wynne feature regularly in this journal and stand out as three of the most influential and consistently stimulating voices in debates about the governance of science. Brian Wynne also provides a link back to the journal’s first issue, as one of its original contributors. Indeed, many of the insights from his original essay (Wynne, 1992), which asked whether we are witnessing “new horizons” or a “hall of mirrors”, remain relevant today. Patrick Sturgis represents the quantitative approaches and methodologies for understanding publics that have long been central to this journal. Other authors bring perspectives from different cultures. David Guston provides a US view, but also reflects on the links he has developed within cultures of practising science. Maja Horst discusses the political cultures within and around the Danish Board of Technology. Michael Burgess describes how engagement activities in Canada both fit within and challenge the institutions of democracy that surround them. Hepeng Jia and Li Liu explain the Chinese situation, in which arguments for and against dialogue are given added urgency by rapid economic and scientific developments. Richard Jones is a British nanoscientist who has, through his policy and public engagement activities over the last decade, become a thoughtful advocate of meaningful dialogue. Helga Nowotny completes the line-up, as not only a prolific scholar and thinker on issues of science and society, but also – as President of the European Research Council – someone who now confronts daily the institutional reality of reconciling scientific, political and public interests.

The involvement of social scientists in the prescription, delivery and evaluation of public engagement with science has been met with the accusation that we are performing a simplistic argument that “the technical is political, the political should be democratic and the democratic should be participatory” (Moore, 2010: 793). If we, as social scientists, are going to continue a normative commitment to the idea of public engagement, we need to develop new lines of argument and analysis. The first thing to acknowledge more openly is that public engagement has typically become a procedural response to a more fundamental political challenge. As Guston argues in this issue, the scale of the challenge – whether expressed as an issue of ‘scientism’, scientific governance or democratisation – dwarfs the small processes of engagement that are put in place. The mini-publics (Goodin and Dryzek, 2006) typically brought together for dialogue exercises look microscopic against the backdrop of global science and its governance. Sturgis and Horst both conclude that we have over-promised on what such public engagement exercises can deliver.

Like other social scientists working in this area, we have at various times designed and run public engagement processes, evaluated them, criticised them and advised policymakers about them. Where it once seemed easy to take sides, to stand up for public engagement against its critics (Taverne, 2005), our experience suggests that such support now needs to be qualified. We have seen at first hand the potential for public engagement to open up (Stirling, 2008) productive and surprising discussions about the politics and purposes of science and we have seen institutions take these seriously. But we have also seen unreflexive public engagement used to close down vital debates in contentious areas. As engagement becomes the norm, the question is perhaps less ‘*why*’ and more ‘*when*’ should we promote public engagement with science? We cannot be the only researchers to have found ourselves recently offering enthusiastic scientists and policymakers advice on how and when *not* to engage.

There needs to be, according to Sturgis, “a transformation to at least rival the one we have witnessed since the inception of this esteemed journal”. With twenty years of experience and discussion on public engagement, perhaps now is the time to step back, widen our gaze and recommit to studying and articulating a more ambitious project of publicly engaged science. This will mean thoughtfully putting public engagement, as conventionally imagined, in its place as part of a broader debate about public value (Wilsdon et al., 2005). It is our hope that the contributions and reflections in this issue will help to stimulate such reflections, and suggest future directions for a conversation about publics, science, democracy and governance that this journal has been at the centre of for the past twenty years. That conversation is more pressing than ever, but must continue to welcome new, more diverse and at times discordant voices to the table.

## Notes

1. Mark Henderson credits the mobilisation of ‘geeks’ or science enthusiasts via social media both for forcing libel reform on to the political agenda in the UK after Simon Singh was sued for libel by the British Chiropractic Association and for helping lobby for no spending cuts in the UK science budget during the 2010 spending review.
